# Heterotrimeric G-protein α subunit (*LeGPA1*) confers cold stress tolerance to processing tomato plants (*Lycopersicon esculentum* Mill)

**DOI:** 10.1186/s12870-020-02615-w

**Published:** 2020-08-26

**Authors:** Xinyong Guo, Juju Li, Li Zhang, Zhanwen Zhang, Ping He, Wenwen Wang, Mei Wang, Aiying Wang, Jianbo Zhu

**Affiliations:** College of Life Science, Shihezi University, Shihezi, 832000 China

**Keywords:** *Lycopersicon esculentum*, *LeGPA1*, Cold tolerance, Overexpression, RNA interference, Antioxidants

## Abstract

**Background:**

Tomatoes (*Lycopersicon esculentum* Mill) are key foods, and their molecular biology and evolution have been well described. Tomato plants originated in the tropics and, thus, are cold sensitive.

**Results:**

Here, we generated *LeGPA1* overexpressing and RNA-interference (RNAi) transgenic tomato plants, which we then used to investigate the function of *LeGPA1* in response to cold stress. Functional LeGPA1 was detected at the plasma membrane, and endogenous *LeGPA1* was highly expressed in the roots and leaves. Cold treatment positively induced the expression of *LeGPA1*. Overexpression of *LeGPA1* conferred tolerance to cold conditions and regulated the expression of genes related to the INDUCER OF CBF EXPRESSION-C-REPEAT-BINDING FACTOR (ICE-CBF) pathway in tomato plants. In the *LeGPA1-*overexpressing transgenic plants, the superoxide dismutase, peroxidase, and catalase activities and soluble sugar and proline contents were increased, and the production of reactive oxygen species and membrane lipid peroxidation decreased under cold stress.

**Conclusions:**

Our findings suggest that improvements in antioxidant systems can help plants cope with the oxidative damage caused by cold stress, thereby stabilizing cell membrane structures and increasing the rate of photosynthesis. The data presented here provide evidence for the key role of *LeGPA1* in mediating cold signal transduction in plant cells. These findings extend our knowledge of the roles of G-proteins in plants and help to clarify the mechanisms through which growth and development are regulated in processing tomato plants.

## Background

Abiotic stresses, such as drought, high temperature, chilling injury, salt injury, and heavy metal toxicity, seriously affect crop growth and food production, resulting in annual losses to agricultural production exceeding $100 billion [1]. To resist injury, plants have evolved physiological pathways for sensing and transmitting transcriptional regulation and responses to low-temperature stress during long-term changes in the natural geographical environment. These include the transmembrane signal transduction pathways, in which signaling components on the cytoplasmic membrane, such as heterotrimeric G-proteins, play a crucial role [2, 3].

Heterotrimeric G-proteins are composed of a complex of three subunits, including Gα, Gβ, and Gγ. The function of G-proteins is well understood in animals [4], and more than 20 species of Gα have been identified in mammals. The Gα subunits have the following functional sites: ADP-ribosylation, GTP/GDP binding, plasma membrane receptor recognition and binding, GTPase activity, and intracellular effector binding sites. These sites are closely related to heterotrimeric G-protein functions, and thus, Gα is generally considered to be a functional subunit [5]. Ma et al. used modern molecular biology techniques based on mammalian G-protein α-subunit sequence homologs to isolate the first *GPA1* gene from *Arabidopsis thaliana*. The protein encoded by the gene has 36% similarity to the mammalian Gα subunit and contains conserved GTP-binding regions [6, 7]. Subsequently, the cDNA sequence of the G-protein α subunit has been isolated in rice [8, 9], wild oat [10], tomato [11], soybean [12, 13], pea [14], spinach [15], and lotus [16]. The predicted amino acid sequences of all the proteins encoded by these cDNAs are similar to the functional domains identified on the mammalian α subunit, suggesting that *GPA1* is a conserved gene ubiquitous to flowering plants. Furthermore, a single copy of *GPA1* occurs in plants, indicating the gene likely has a non-redundant function in plant cells.

Heterotrimeric G-proteins are involved in several growth and development processes in plants. For example, overexpression of *GPA1* can increase the sensitivity of Arabidopsis to gibberellin, resulting in increased response to gibberellin during seed germination [17–19], which further affects germination. Gα can affect root development and positively regulate the growth and development of lateral roots [20]. At the same time, Gα can control the growth and development of the hypocotyl of seedlings by regulating cell differentiation [21, 22]. Compared with *Arabidopsis*, Gα-coding-gene deletion mutants of rice had a wider and darker leaf color phenotype and shorter plant size [23, 24]. Studies have shown that the number of cells in *dl-l* mutants is decreased [25]. Therefore, Gα plays a positive regulatory role in rice cell differentiation.

Heterotrimeric G-proteins are also key signal transduction regulators in plants, controlling pathways related to abiotic stress [26]. In Arabidopsis, *GPA1* is involved in oxidative stress signal transduction and can positively regulate the abiotic stress factors upstream of reactive oxygen species (ROS) and nicotinamide adenine dinucleotide phosphate (NADPH) [27]. Arabidopsis Gα subunit mutations reduced the sensitivity of the plant to ozone stress [28]. In pea (*Pisum sativum* Linn.), constitutive overexpression of Gα subunits enhanced transgenic pea resistance to salt stress [29]. Studies by Ferrero-serrano and Assmann have shown that under drought stress, the *d1* mutant Gα subunit of rice has a lower leaf temperature than the wild type and, thus, stronger resistance to drought stress [30]. Chakraborty et al., in their work on a group of Arabidopsis Gα subunit mutants, have reported that abiotic-stress-related gene expression is altered, suggesting that *GAP1* is involved in *Arabidopsis* responses to heat and cold stress [31, 32]. The expression of rice Gα, Gβ, and Gγ subunits was also strongly induced by cold stress, which suggests these subunits have an active regulatory role in cold stress resistance in rice [33, 34]. Studies by Ma et al. have shown that, in rice under cold stress, the Gα subunit RGA1 (Rice G protein α subunit 1) interacts with CHILLING TOLERANCE DIVERGENCE 1 (COLD1) on the plasma membrane and endoplasmic reticulum, activating Ca^2+^ channels and enhancing resistance to low temperatures [35].

The processing tomato (*Lycopersicon esculentum* Mill.) was first grown in subtropical and tropical regions, but is now cultivated worldwide. These tomato plants are highly susceptible to cold stress, which thus has the potential to inflict substantial economic damage [36]. Therefore, identifying key genes for low-temperature tolerance is of great theoretical and practical significance.

To reveal the mechanisms behind the molecular regulation of low-temperature tolerance in tomato plants and to cultivate new low-temperature tolerant varieties, the *GPA1* gene was cloned from processing tomatoes. Using a transgenic approach, we constructed overexpression and RNA-interference vectors for the *LeGPA1* gene and studied the roles of *LeGPA1* in plant growth and development and the low-temperature response. This study led to the genetic engineering of a novel series of low-temperature tolerant tomato plants. These low-temperature tolerant plants are now a valuable asset in the efforts to reveal the molecular regulation mechanisms of low-temperature resistance in processing tomato plants.

## Results

### Bioinformatics analysis of *LeGPA1*

**Fig. 1:**
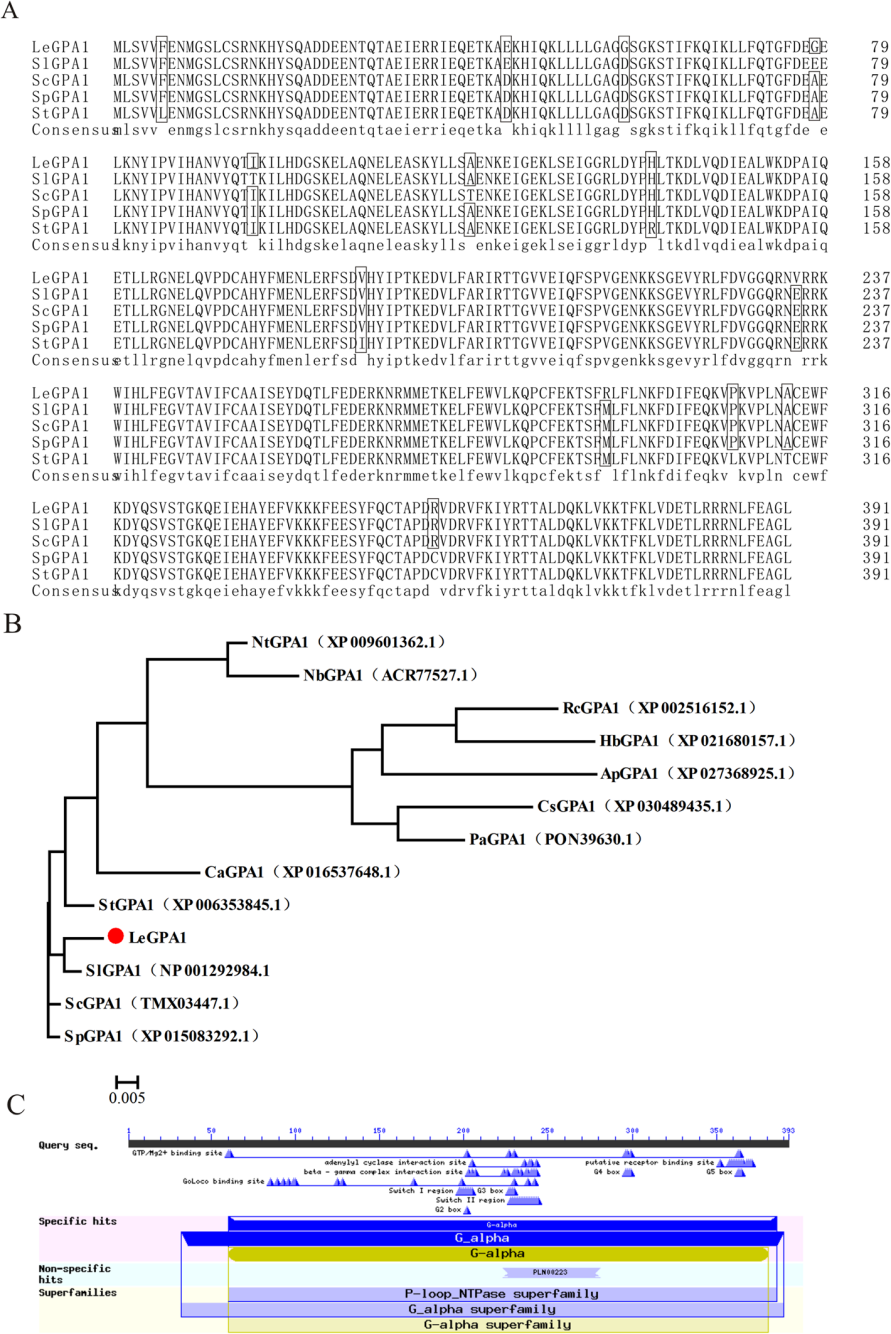
*Lycopersicon esculentum* LeGPA1 sequence analysis*.* **a** LeGPA1 amino acid sequence alignment with other plant species. **b** Phylogenetic relationship between LeGPA1 protein and GPA1 proteins from other plant species. MEGA 5.1 was used for phylogenetic tree construction, with protein sequences used for such construction being from GenBank as follows: *Nicotiana tomentosiformis* (XP_009601362.1); *Nicotiana benthamiana* (ACR77527.1); *Ricinus communis* (XP_002516152.1); *Hevea brasiliensis* (XP_021680157.1); *Abrus precatorius* (XP_027368925.1); *Cannabis sativa* (XP_030489435.1); *Trema orientale* (PON39630.1); *Capsicum annuum* (XP_016537648.1); *Solanum tuberosum* (NP_001275141.1); *Solanum lycopersicum* (NP_001292984.1); *Solanum chilense* (TMX03447.1); and *Solanum pennellii* (XP_015083292.1). Bootstrap replicate values (1000×) were obtained. **c** Prediction of the conserved domain of LeGPA1

Using cDNA from processing tomato leaves as a template and gene specific primers, we amplified the *LeGPA1* gene (1176 bp) by PCR. The *LeGPA1* gene encodes 392 amino acids. DNAMAN software was used to compare LeGPA1 and other plant GPA1 protein amino acid sequences. We found that the LeGPA1 protein from processing tomato had the highest similarity (98.72%) with the amino acid sequence of the common tomato (*Solanum lycopersicum*) (Fig. 1a). Only single amino acid point mutations were detected, and there are no insertions or deletions. Phylogenetic trees of the LeGPA1 sequences from 13 species were established by ClustalX 2 and MEGA 4.1 software. We found that the LeGPA1 of processing tomato and SlGPA1 of the common tomato are on the same phylogenetic branch, and therefore, are closely related (Fig. 1b). The Conserved Domains tool of NCBI was used to analyze the conserved domains of sequences encoded by the *LeGPA1* gene. The protein encoded by this gene has a guanine nucleotide-binding protein subunit alpha domain, which belongs to the G-alpha family (Fig. 1c).

### Subcellular localization of LeGPA1

**Fig. 2:**
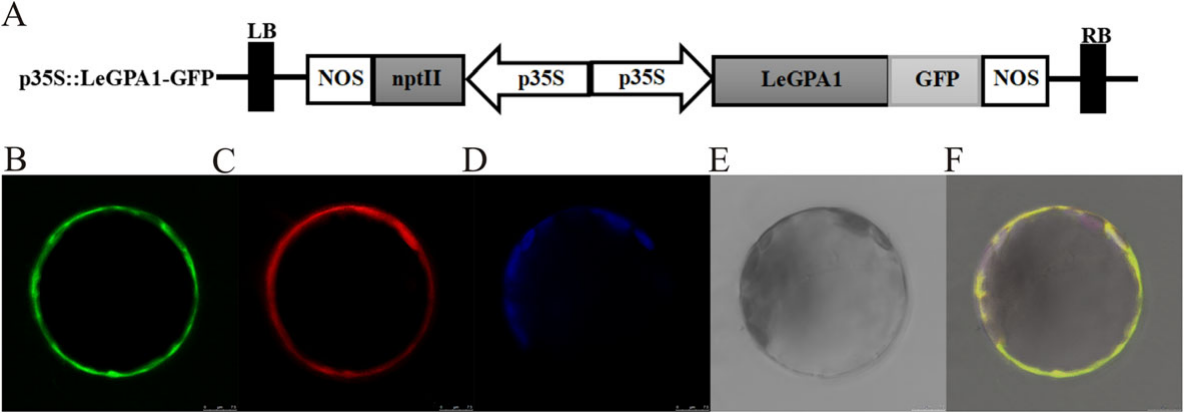
LeGPA1 localization within cells. Arabidopsis mesophyll protoplasts were co-transformed with p35S-*LeGPA1*-GFP and a plasma membrane marker PM-rk. We co-transformed Arabidopsis mesophyll protoplasts using p35S-*LeGPA1*-GFP and the PM-rk plasma membrane marker. **a** The p35S-*LeGPA1*-GFP plasmid. **b** Arabidopsis mesophyll protoplasts following *LeGPA1*-GFP fusion protein expression. **c** Arabidopsis mesophyll protoplasts following Pm-rk expression. **d** Chloroplasts within Arabidopsis protoplasts. **e** A bright-field image of Arabidopsis mesophyll protoplasts. **f** Merged fluorescent images collected from *LeGPA1*-GFP and PM-rk expressing protoplasts. Scale bars = 7.5 μm

To determine the location of LeGPA1 in cells, the *LeGPA1* gene was cloned into a pCAMBIA2300-35S-GFP (green fluorescent protein) vector downstream of the 35S promoter and upstream of the GFP gene. The fusion protein expression vector p35S-*LeGPA1*-GFP constitutively expressed LeGPA1-GFP (Fig. 2a). The plasmids were extracted, and p35S-*LeGPA1*-GFP and pm-rk (cell membrane marker) were transformed into *Arabidopsis* protoplasts. By this approach, LeGPA1 was found to localize to the cell membrane (Fig. 2b).

### Expression analysis of *LeGPA1* in processing tomato

**Fig. 3:**
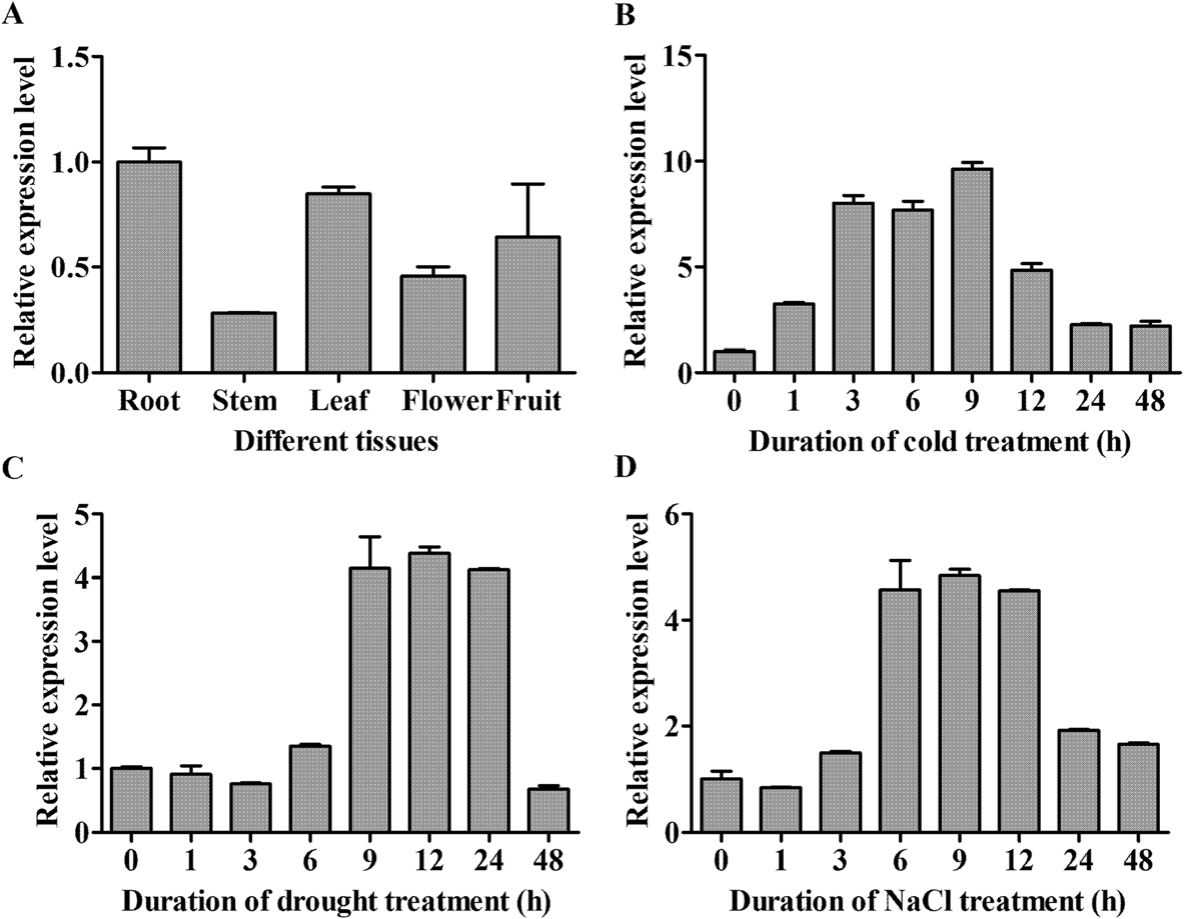
Assessment of *LeGPA1* mRNA expression in wild-type plants. **a** *LeGPA1* expression was evaluated in the fruits, stems, leaves, flowers, and roots of *L. esculentum* following growth at 25 °C. **b** *LeGPA1* gene expression was evaluated after 0, 1, 3, 6, 9, 12, 24, and 48 h at 4 °C in the leaves of these plants. **c** Expression of the *LeGPA1* gene in *L. esculentum* leaves following drought stress generated using 20% PEG-6000 for 0, 1, 3, 6, 9, 12, 24, and 48 h. **d** Expression of *LeGPA1* gene in leaves of *L. esculentum* treated with salt stress generated using 200 mM NaCl for 0, 1, 3, 6, 9, 12, 24, and 48 h. Reported data are derived from triplicate analyses

By quantitative reverse transcription PCR (qRT-PCR), we analyzed the *LeGPA1* expression patterns in the various organs of wild-type processing tomato. Detectable *LeGPA1* expression was found in all examined organs, with maximal expression in roots, then leaves, and fruits, and the lowest expression in stems (Fig. 3a). To further verify whether the expression of *LeGPA1* is induced by stress, we also measured *LeGPA1* expression under different stress treatments. As shown in Fig. 3b, the low-temperature treatment rapidly induced the upregulation of *LeGPA1* gene within 3 h by 8.01 times. Then the gene expression level slowly decreased to 7.69 times of that before the treatment during the next 3 h, while reaching the peak value at 9 h treatment (9.63 times higher). These indicate that low-temperature treatment could upregulate *LeGPA1* gene expression. When treated with 20% PEG-6000, the alteration of gene expression during the first 6 h was very slow. Whereas, at 9 and 12 h, the expressions of *LeGPA1* were 4.14 and 4.38 (maximum) times than that before treatment, respectively, followed by a slow decrease at 24 h and then a rapid decrease afterwards (Fig. 3c). The treatment of 200 mm NaCl upregulated the expression level of *LeGPA1*, with the maximum effect achieved at 9 h (4.84 times higher) and a rapid decrease for level-off after 12 h (Fig. 3d). Based on these results, we concluded that *LeGPA1* could be induced by a variety of stresses.

### Identification of transgenic processing tomatoes

**Fig. 4:**
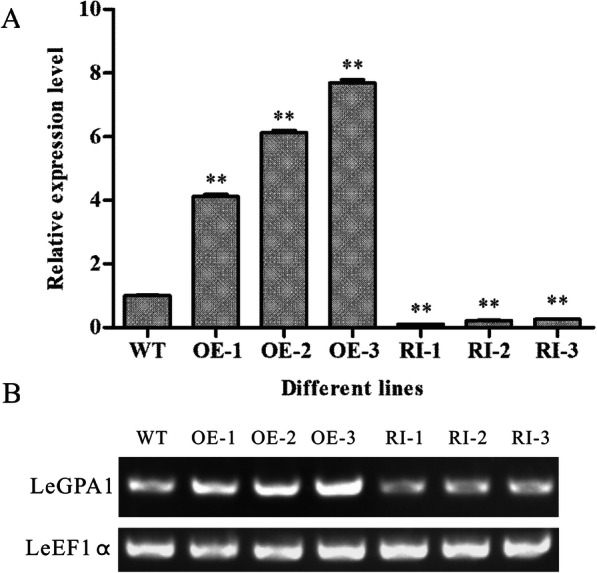
*LeGPA1* expression in wild-type (WT), *LeGPA1*-overexpressing transgenic tomato plants (OE1-OE3), and RNAi-expressing transgenic tomato plant lines (RI1-RI3). **a** qRT-PCR was used to assess gene expression. **b** Semi-quantitative PCR was used to assess expression in leaves collected from plants grown under standard conditions. Experiments were repeated twice, with results all being representative of triplicate samples. Bars represent SDs. *P < 0.05 and **P < 0.01

To evaluate the importance of *LeGPA1* in the low-temperature stress response of processing tomato, we used an Agrobacterium-mediated method to generate transgenic plants (*LeGPA1*-overexpressing and gene silencing) (Fig. S1). Regenerated plants were obtained and tested by PCR. Three *LeGPA1-*overexpressing transgenic lines from positive plants (OE-1/2/3) and three RNA-interfering transgenic lines (RI-1/2/3) were selected and used for qRT-PCR detection (Fig. 4). Relative *LeGPA* mRNA levels in OE-1, OE-2, and OE-3 plants increased 4.12-, 6.13-, and 7.69-times, respectively, compared with wild-type plants, and *LeGPA* mRNA levels in RI-1, RI-2, and RI-3 decreased by 0.90-, 0.78-, and 0.74-times, respectively. Therefore, based on the results of gene expression analysis, we selected two overexpression lines (OE-2, OE-3) and two RNA-interfering lines (RI-1, RI-2) for further research on cold-resistance function.

### Analysis of biological characteristics of transgenic tomato

**Fig. 5:**
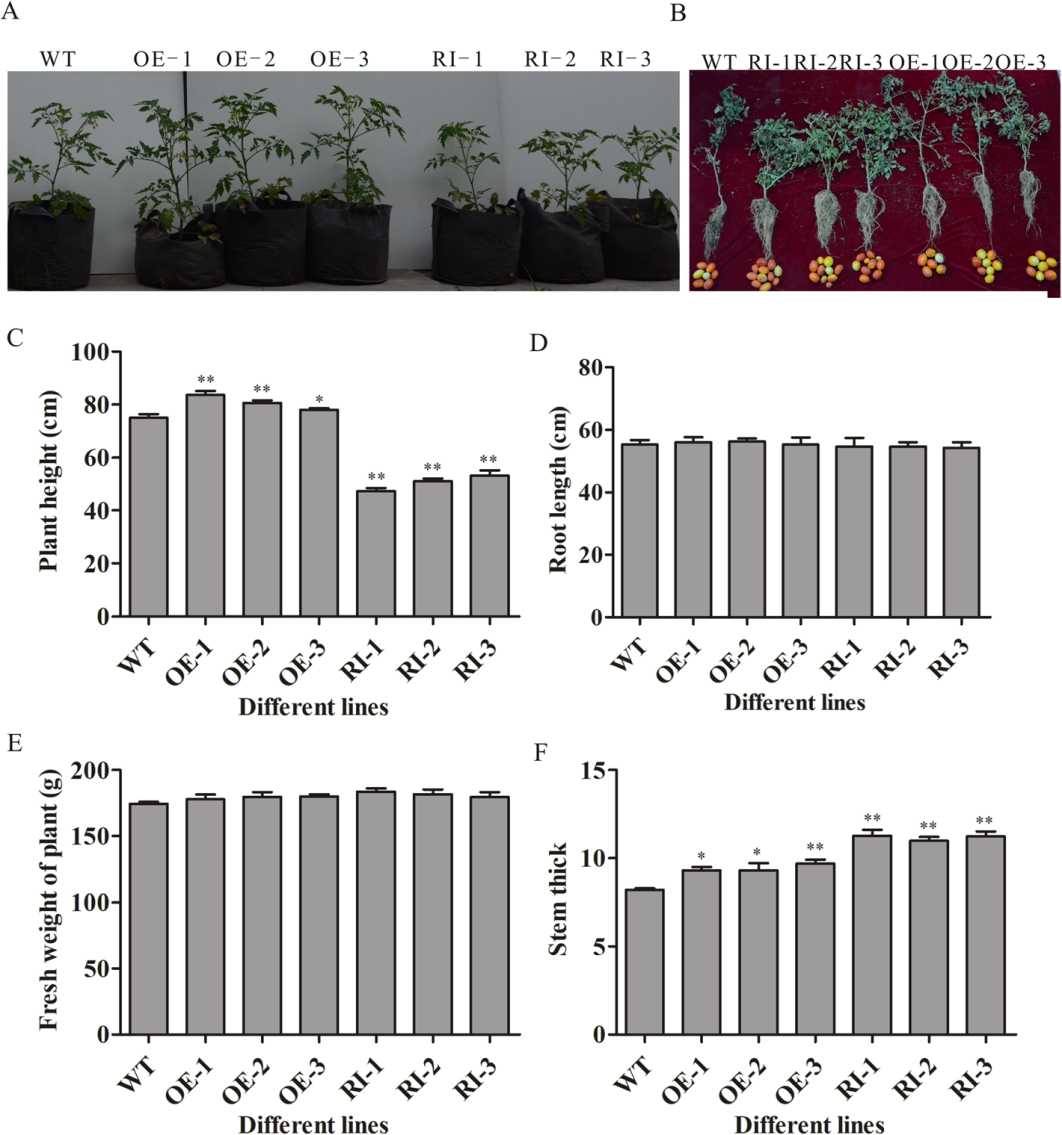
Assessment of the characteristics of wild-type and transgenic plants. **a** The growth of wild-type plants and transgenic plants expressing the *LeGPA1* gene in the field. **b** Plant fruiting. **c** Plant height. **d** Root length. **e** Plant fresh weight. **f** Stem thick. Data are the means and SDs from triplicate samples. *P < 0.05 and **P < 0.01 vs. WT.

We tested a panel of biological indicators (plant height, stem thickness, root length, and root fresh weight) among 80-day-old plants, comparing the wild-type and transgenic tomatoes. As shown in Fig. 5, We found that the *LeGPA1-*overexpressing transgenic tomato plants grew significantly taller compared with wild-type controls, by 4–11.6%. However, the plant growth of RNA interference lines was lower than that of wild-type tomatoes, by 28.9–36.9%. Root length or fresh weight were comparable in wild-type and transgenic plants. In contrast, the stem thickness of the *LeGPA1-*overexpressing transgenic plants increased by 13.5–18.3% compared with wild-type plants, while the stem thickness of the RNA-interference transgenic plants was significantly increased (by 34.1–37.4%) compared with wild-type controls (*P* < 0.01).

### Overexpression of the *LeGPA1* gene enhanced the resistance of transgenic tomato seedlings to low temperature

**Fig. 6:**
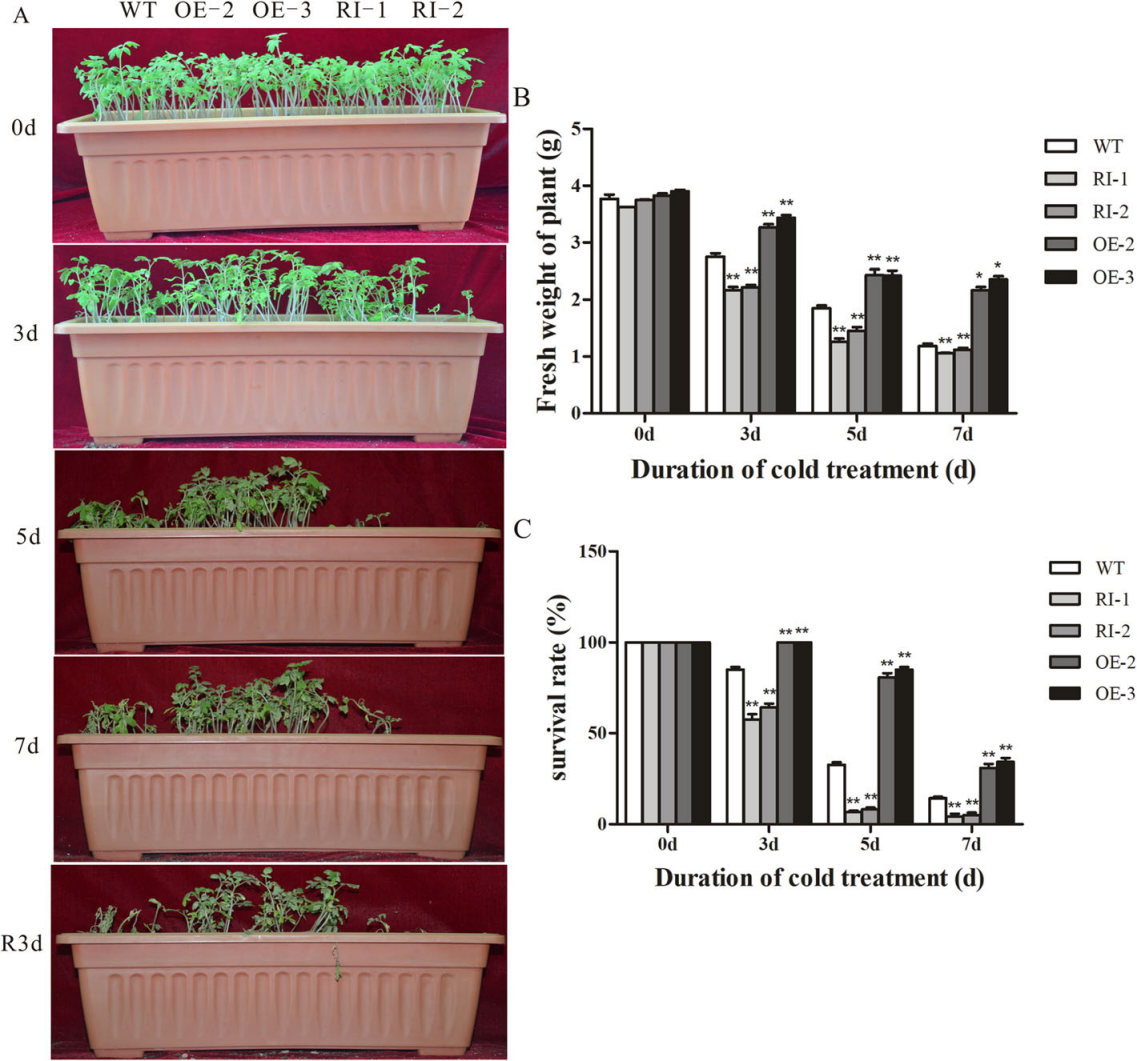
Assessment of young wild-type (WT) and transgenic tomato plant cold resistance. **a** Phenotypes of untreated 3-week-old WT and transgenic tomato plants or those treated with cold stress for 5 d. **b** Fresh weight. **c** Survival rate. Data are the means and SDs from triplicate samples. *P < 0.05 and **P < 0.01

To test the cold stress tolerance of the transgenic tomato plants, T2-generation seeds of *LeGPA1*-overexpressing transgenic plants, RNA-interference transgenic plants, and wild-type processing plants were uniformly seeded into rectangular pots. The plants were grown under normal conditions (25 °C) for 3 weeks and then transferred to a 4 °C incubator for 7 days. Phenotypes were observed, and seedling fresh weight and survival were measured at 0, 3, 5, and 7 days at low temperature. As shown in Fig. 6a, both wild-type and transgenic plants grew normally at 4 °C. After 3 days of 4 °C treatment, RNA interference tomato seedlings began to show signs of wilting (slight curving of plant parts was seen). Wild-type tomato seedlings were slightly wilted but not as much as the RNA-interference lines, while the *LeGPA1*-overexpressing tomato plant seedlings showed better growth than the RNA-interference seedlings. After 5 days at low temperature, most of the RNA interference tomato seedlings had bent and fallen, the wild-type processing plants began to have curved tops that inhibited growth, and some of the *LeGPA1*-overexpressing lines also showed wilting. After 7 days of treatment, the RNA-interference seedlings had almost all perished, with only a few surviving; there were few surviving wild-type processing seedlings but more than the surviving RNA-interference lines, while the *LeGPA1* transgenic seedlings had only undergone partial wilting. After 7 days of low-temperature treatment, the tomato seedlings were placed in a 4 to 25 °C incubator for 3 days under normal conditions to recover. It was found that almost all the RNA-interference tomato seedlings had died, a few wild-type plants survived, and a few *LeGPA1*-overexpressing plants had died. Fresh weight measurements and survival statistics were also performed, and similar results were obtained. Following 7 days of cold treatment, the fresh weight of the *LeGPA1*-overexpressing lines had decreased by 43.4% (OE-2) and 39.6% (OE-3), that of the wild-type plants had decreased by 68.5%, and that of the RNA-interference lines had decreased by 70.7% (RI-1) and 70.1% (RI-2), relative to the values taken before treatment (Fig. 6b). Following a 7-day low-temperature treatment period, both transgenic and wild-type seedlings exhibited significantly decreased survival. However, the survival rate of the *LeGPA1*-overexpressing plants was increased significantly compared with the RNA-interference plants and wild-type controls, and the wild-type controls had higher survival rates compared with RNA-interfered plants (Fig. 6c). These data show that overexpression of *LeGPA1* increased transgenic tomato seedling resistance to low temperatures.

### Growth analysis of transgenic tomato sprouts under low-temperature stress

**Fig. 7:**
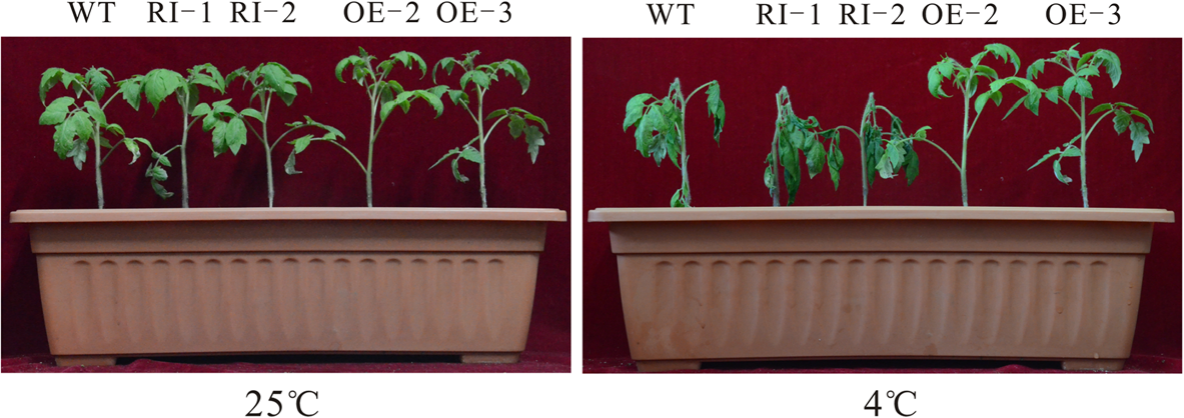
The growth of 6-week-old WT and transgenic plants for 0 or 5 days under 4 °C

To further assess transgenic tomato low-temperature resistance, transgenic and wild-type plants were next grown for 6 weeks under standard conditions before being transferred for 5 days to 4 °C. Normal growth in wild-type and transgenic plants was observed at room temperature. Following growth at 4 °C, the wild-type tomato leaves had wilted to different degrees, while all the RNAi tomato leaves had wilted and drooped, were dark brown with water stains on the surface, and some of the leaves had died. However, there was almost no change to the *LeGPA1*-overexpressing tomatoes, which grew normally (Fig. 7). This indicates that *LeGPA1* is an important mediator of low-temperature resistance within these processing tomatoes.

### Overexpression of *LeGPA1* alleviates cell membrane damage under low-temperature stress

**Fig. 8:**
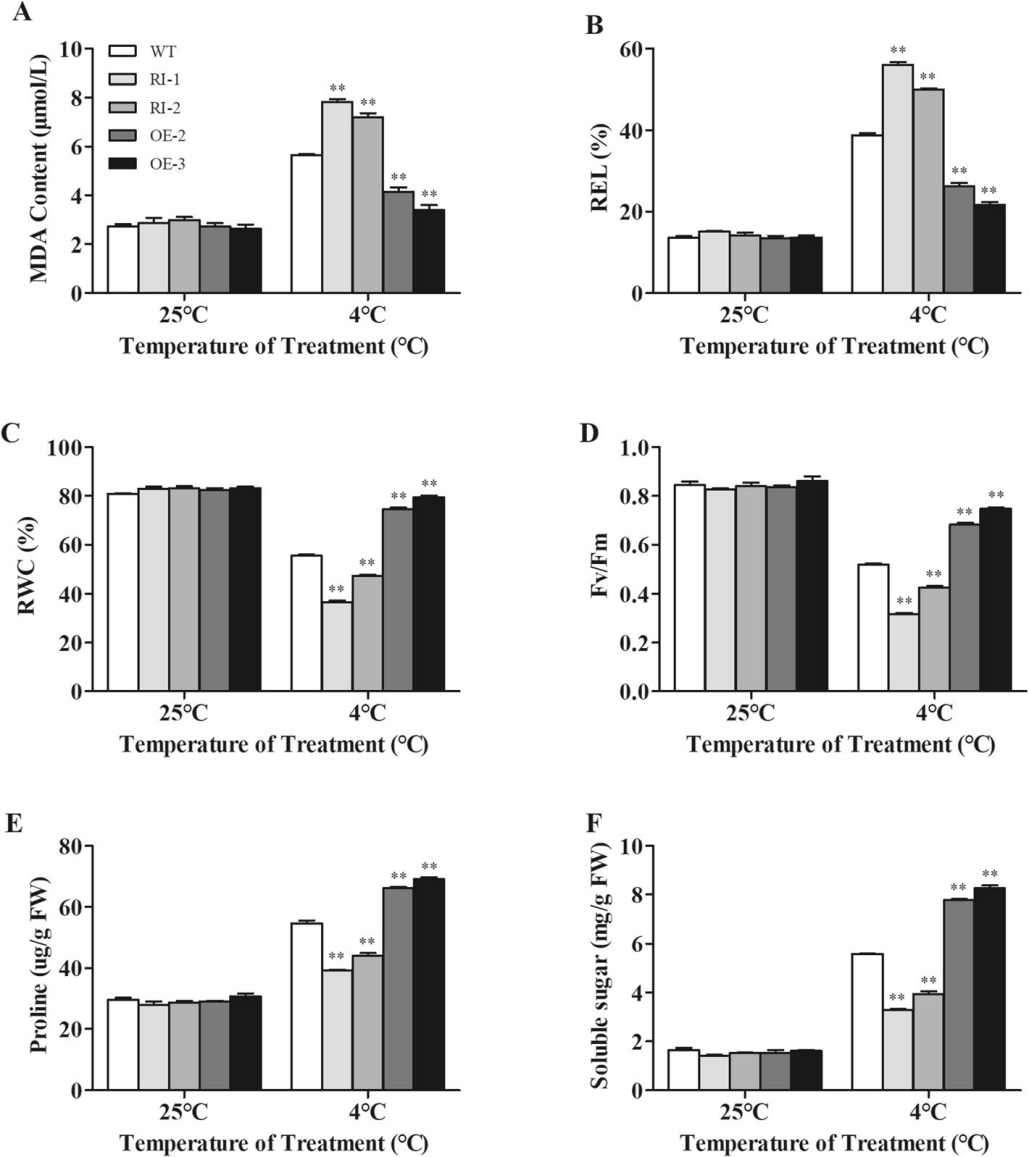
Changes in WT and transgenic plant responses to cold stress in 6-week-old plants. **a** MDA contents. **b** REL. **c** RWC. **d** F_v_/F_m_ values. **e** Proline contents. **f** Solute sugar levels. Uniformly sized 6-week-old tomato plants were subjected to a 5-day cold stress exposure at 4 °C. Leaves that were the second and third from the top were assessed. Data are the means and SDs from triplicate samples. *P < 0.05 and **P < 0.01

Malondialdehyde (MDA) is a ROS-associated lipid peroxidation byproduct. Cell membrane permeability can be gauged based on MDA levels, as well as relative electrolyte leakage (REL). Under low-temperature stress, the MDA and REL levels of both wild-type and transgenic plants rapidly increased (Fig. 8a, b). The MDA and REL levels of RNA-interference plants were elevated compared with wild-type controls. However, wild-type plants exhibited markedly elevated MDA and REL levels relative to *LeGPA1*-overexpressing transgenic plants (*P* < 0.01).

The relative water content (RWC) of plants reflects their water retention capacity and is used as a measure of plant water status and osmotic regulation. The RWCs of the transgenic and wild-type leaves were comparable to those before low-temperature stress (Fig. 8c). In contrast, the RWCs of both plant types decreased after stress, but the decline in the RWC of wild-type controls was significantly greater relative to that of the *LeGPA1*-overexpressing transgenic plants (*P* < 0.01). The RWC values of the RNA-interference plants were markedly higher when compared with the wild-type controls (P < 0.01).

The maximum photochemical efficiency of photosystem II (PSII) was calculated as Fv/Fm. Under normal conditions, we detected no significant differences in photochemical efficiency when comparing the wild-type and transgenic plants (Fig. 8d). However, at low temperatures, the photochemical efficiencies of the wild-type and transgenic lines differed significantly (P < 0.01). In all plants, the value steadily declined, but the decline was greater in magnitude in the wild-type plants relative to that of the *LeGPA1*-overexpressing transgenic plants, and still greater in magnitude in the RNA-interference lines.

Under low-temperature stress (vs. normal ambient growth temperature), all plants had increased soluble sugar and proline contents. Compared with wild-type plants, the *LeGPA1*-overexpressing transgenic plants had higher levels of soluble sugar and proline, and the RNA-interference transgenic plants had lower soluble sugar and proline levels (Fig. 8e, f). These findings suggest that the overexpression of *LeGPA1* in processing tomato plants protects against the damage caused by low-temperature stress.

### Overexpression of *LeGPA1* alleviates the accumulation of ROS in response to low-temperature stress

**Fig. 9:**
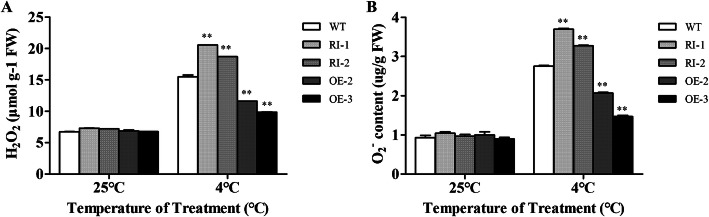
ROS (H_2_O_2_ and O_2_^−^) accumulation in WT and transgenic tomato plants exposed to cold stress. **a** H_2_O_2_ levels. **b** O_2_^−^ content. Uniformly sized 6-week-old tomato plants were subjected to a 5-day cold stress exposure at 4 °C. Leaves that were the second and third from the top were assessed. Data are the means and SDs from triplicate samples. *P < 0.05 and **P < 0.01

Before applying a low-temperature treatment, the H_2_O_2_ and O_2−_ contents were low and almost identical in the wild-type and transgenic plants (Fig. 9). However, the ROS levels in all tested plants increased after exposure to low-temperature stress. Compared with wild-type plants, this increase was significantly greater in the RNAi plants and less in the *LeGPA1***-**overexpressing plants. We found that overexpression of *LeGPA1* reduced the accumulation of H_2_O_2_ and O_2_^−^ under low-temperature stress.

**Fig. 10:**
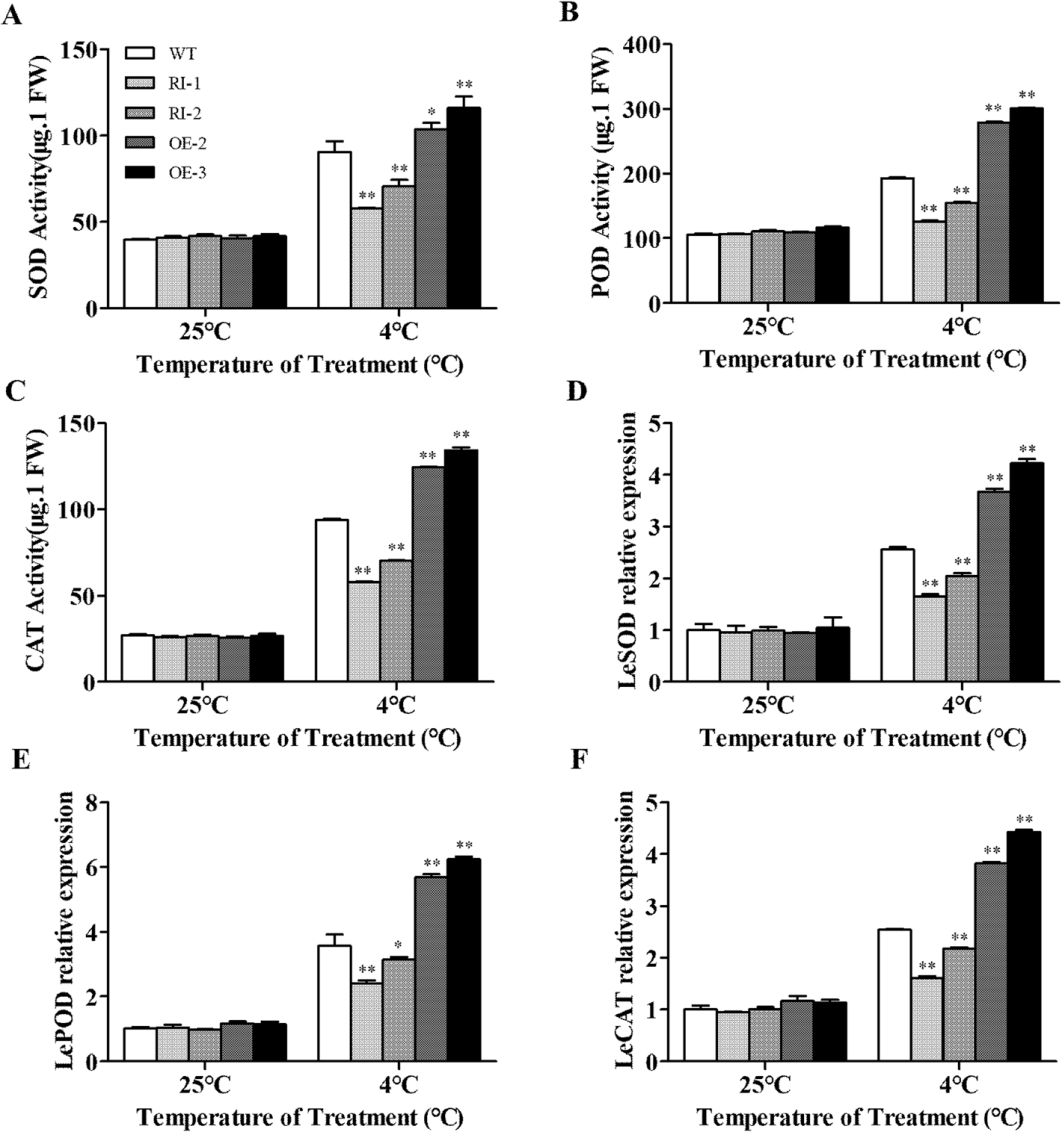
Antioxidant enzyme (SOD, POD, and CAT) activity and expression of ROS-scavenging genes in WT and transgenic tomato plants exposed to cold stress. **a** SOD activity. **b** POD activity. **c** CAT activity. **d** Expression level of *LeSOD*. **e** Expression level of *LePOD*. **f** Expression level of *LeCAT*. Uniformly sized 6-week-old tomato plants were subjected to a 5-day cold stress exposure at 4 °C. Leaves that were the second and third from the top were assessed. Data are the means and SDs from triplicate samples.*P < 0.05 and **P < 0.01

To study the effects of overexpression and silencing of *LeGPA1* on low-temperature stress-related oxygen scavenging, superoxide dismutase (SOD), peroxidase (POD), and catalase (CAT) enzyme activities were measured in wild-type and transgenic plants. We found that following low-temperature exposure, these three activities increased in all plants. However, relative to wild-type plants, there were significantly higher CAT, POD, and SOD activities in the *LeGPA1***-**overexpressing transgenic plants (*P* < 0.01). The SOD, CAT, and POD activities in wild-type plants were significantly higher than those in RNA-interference plants (Fig. 10a-c). We found that overexpression of the *LeGPA1* gene increased the activity of oxygen scavenging enzymes under low-temperature stress, improving the plant’s ROS scavenging ability and reducing ROS toxicity. This suggests that transgenic tomato plants can degrade more ROS. To determine the cause of the high CAT, SOD, and POD activities in transgenic tomato plants, we measured the *LeSOD*, *LePOD,* and *LeCAT* transcript levels. In standard growth conditions, the *LeSOD*, *LePOD*, and *LeCAT* expression levels were similar in all lines. Following low-temperature exposure, we recorded the upregulation of *LeSOD*, *LePOD*, and *LeCAT* in all plants, and the expression levels in the *LeGPA1***-**overexpressing lines were higher compared with wild-type lines. However, the levels in the RNA-interference transgenic tomatoes were low (Fig. 10e-f). Therefore, we conclude that the high levels of *LeSOD*, *LePOD*, and *LeCAT* expression increased the corresponding enzyme activity and cleared more H_2_O_2_ and O_2_^−^ ROS in the *LeGPA1***-**overexpressing transgenic tomatoes.

### *LeGPA1* has a positive regulatory effect on cold-response gene expression in processing tomatoes

**Fig. 11:**
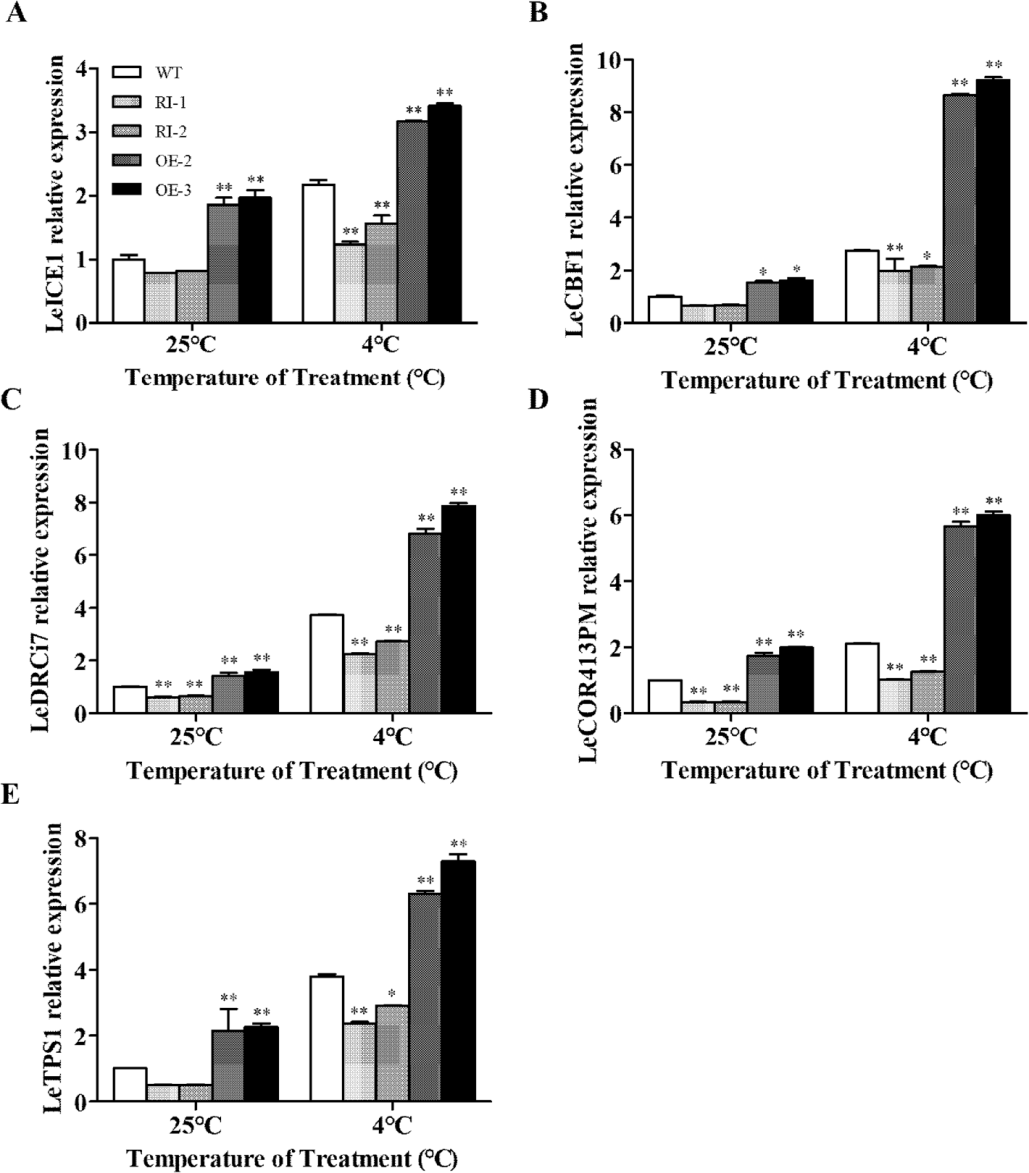
Relative expression of stress-related genes in WT and transgenic tomato plants exposed to cold stress. **a** Expression level of *LeICE1*. **b** Expression level of *LeCBF1*. **c** Expression level of *LeDRCi7.* **d** Expression level of *LeCOR413PM.* **e** Expression level of *LeTPS1.* Total RNA was extracted from untreated and cold-treated (4 °C) 6-week-old WT and transgenic tomato plants. The expression levels of stress-related genes were analyzed by qRT-PCR. Data are the means of three replicate samples. Bars represent SDs. *P < 0.05 and **P < 0.01

We determined the expression levels of the INDUCER OF CBF EXPRESSION (ICE) and C-REPEAT-BINDING FACTOR (CBF) encoding genes (*LeICE1* and *LeCBF1*) and their downstream target genes (*LeTPS1*, *LeCOR413PM2*, and *LeDRCi7*) in processing tomato lines. We found that before low-temperature stress, the RNAi lines exhibited reduced gene expression compared with wild-type plants, and the differences in the expression levels of *LeCOR413PM2* and *LeDRCi7* were highly significant. However, the expression levels of the genes were significantly lower in the wild-type than in the overexpressed lines. Following low-temperature stress, the gene expression levels of all plants showed an overall increasing trend; however, the difference in expression between wild-type and *LeGPA1***-**overexpressing lines further increased and showed extremely significant differences (Fig. 11).

## Methods

### Plants

Wild-type *L. esculentum* (‘Yaxin 87–5’) seeds were obtained from Yaxin Seed Co. Ltd. (Shihezi City, Xinjiang, China). Plants were first grown in a 25 °C tissue culture room (16/8 h light/dark cycle) at 60–70% relative humidity and 200 μmol m^− 2^ s^− 1^ light intensity. Seedlings were next transplanted to pots that contained an equal mixture of soil, peat, and vermiculite, and were transferred to a 22–28 °C greenhouse with natural lighting and identical humidity and light cycle conditions. While in this greenhouse, plants were irrigated with 500 mL of Hoagland’s nutrient solution two times each week.

### Assessment of the impact of stress on *LeGPA1* gene expression

Seeds of wild-type tomato were sown in plastic pots. Then, the seedlings were grown under 14-h light/10-h dark photoperiod at 25 ± 3 °C temperature and 60–70% relative humidity in a controlled greenhouse. By qRT-PCR, we assessed *LeGPA1* gene expression in a range of organs (fruit, flowers, stems, leaves, and roots) from 80-day-old wild-type tomato plants. Also, *LeGPA1* expression was assessed in wild-type tomatoes under cold, salt, or drought stress, carried out under the same conditions as above. All the stress treatments were performed using potted plants at the stage of 6–7 fully expanded leaves, 6-weeks-old, wild-type tomatoes seedlings selected based on their uniformity. For cold stress, wild-type tomato seedlings were well watered and then randomly placed in a cold chamber at 4 °C for 48 h; control seedlings were placed under the same conditions but at 25 °C. For drought conditions, wild-type tomato seedlings were removed from the soil and then divided into two groups: the drought treatment group and the control group. The roots of seedlings in the drought treatment group were submerged 5-cm deep in 20% PEG 6000 for 48 h. The roots of the control group seedlings were submerged 5-cm deep in water for 48 h. Salt stress was induced by removing wild-type tomato seedlings from their soil and immersing the roots in 200 mM NaCl for 48 h. The control group seedlings roots were submerged in water for 48 h. Leaves from identical positions on these plants were collected at 0, 1, 3, 6, 9, 12, 24, and 48 h after the initiation of stress conditions. Plants were grown in three separate growth chambers for replicate samples. Collected leaves were snap-frozen before storage at − 80 °C.

An RNAprep Pure Plant Kit (Tiangen, China) was used to isolate sample RNA for qRT-PCR, after which cDNA was synthesized and analyzed with SYBR Green I Master Mix using a LightCycler 480II platform (Roche Biochemicals, Indianapolis, IN, USA). As a normalization control for cold stress-related gene expression, the tomato *EF1* gene (GenBank ID: X53043) was utilized [37]. Thermocycler settings were: two cycles of 95 °C for 30 s; 50 cycles of 95 °C for 5 s, 60 °C for 10 s, and 68 °C for 10 s. The 2^-ΔΔCt^ method [38] was used to measure the relative gene expression in stress treated plants compared with control plants. The primers used for this assay are shown in Supplementary Table S1. Three biological and technical replicates have been used during real-time PCR analysis. To ensure that a single discrete species was amplified, a melt curve analysis was performed at the end of the real-time PCR.

### *LeGPA1* sequence assessment

DNAMAN (v8.0) was used to align the *LeGPA1* sequence. The TMHMM algorithm (http://www.cbs.dtu.dk/services/TMHMM/) was used for predicting transmembrane domains. MEGA 5.1 (http://www.megasoftware.net/) was used for phylogenetic analyses based on a Neighbor-Joining approach, and 1000 bootstrap replicates, with the deletion of bootstrap scores of < 50%.

### *LeGPA1* cloning

An RNAisoPlus kit (TaKaRa, Dalian, China) was used to extract RNA from the leaves of tomato plants, with DNase I on-column digestion being conducted based on provided instructions. PrimeScript RTase (TaKaRa, Dalian, China) was used for first-strand cDNA synthesis, after which PCR was used to amplify full-length *LeGPA1* using the *LeGPA1* (*Kpn* I)-CF and *LeGPA1* (*Sal* I)-C*R* (Table 1) primers designed using *Solanum lycopersicum* gene sequences (GenBank ID: NM_001306055.1). PrimeSTAR Max DNA polymerase (TaKaRa, Dalian, China) was used for PCR, with the following thermocycler settings: 95 °C for 5 min; 35 cycles of 95 °C for 30 s, 56 °C for 30 s; 72 °C for 10 min. This approach yielded a 1176-bp PCR fragment, which was then cloned into the pMD19-T vector (TaKaRa, Dalian, China). The identity of this fragment was confirmed via DNA sequencing.

### Plasmid construction for overexpression and RNA interference (RNAi)

PCR-amplified fragment of *LeGPA1* was cloned into pCAMBIA2300 with *Kpn*I and *Sal*I restriction sites for *LeGPA1* overexpression, for which the transcription of *LeGPA1* was driven by the 35S promoter. The RNAi construct was built by inserting a PCR-generated 220-bp segment of *LeGPA1* cDNA (segment 340–560 bp) (the RNAi target) into pGM-T plasmid, followed by transformation into *E. coli* DH5α. The correct positive clone was named pGM-S (Supplemental Table S1). This pGM-S construct was then restriction enzyme digested (*Xho*I and *Bgl*II), and the smaller fragment was recovered and ligated with linearized (*Xho*I and *Bgl*II digested) pUCCRNAi. The recombinant pUCS1 vector was collected from *E. coli* DH5α transformed with the ligated product. The pGM-S was then digested by restriction enzymes (*Xho*I and *Bgl*II) for subsequent small fragment recovery. The pUCS1 was digested by restriction enzymes (*Sal*I and *BamH*I) for subsequent large fragment recovery. A reverse repeat sequence of the intermediate vector pUCS1S2 was generated by using pUCCRNAi intron. Then, pUCS1S2 was digested with *Pst*I, followed by recovering the small fragments and cloning it into a linearized pCAM2300 vector (digested by *Pst*I). Similarly, a reverse repeat sequence of the plant RNAi expression vector pCAS was also obtained. Finally, the pCAS was transferred into Agrobacterium GV3101 through freeze-thawing.

### Analyses of the subcellular localization of LeGPA1

The complete open reading frame (ORF) of *LeGPA1* excluding the stop codon was PCR-amplified with the primers containing *Xba*I and *BamH*I restriction site sequences, listed in Table 1. The p35S-*LeGPA1*-GFP was generated by cloning the amplicon into pCAMBIA2300-GFP vector, followed by transducing into *Arabidopsis* mesophyll protoplasts using polyethylene glycol. Similarly, to examine the plasma membrane localization, the PM-rk plasmid was transduced following the same approach [39–41]. The protoplasts were cultured at 23 °C for 16 h, and then a confocal microscopy (Leica Microsystems, Germany) was used to visualize the fluorescence at 488, 561, and 633 nm excitation wavelengths.

### Transgenic plant preparation

Transgenic plants that overexpressed either *LeGPA1* or an RNAi construct were prepared for this study. Briefly, under aseptic conditions, wild-type tomato seeds were evenly spread on ½-MS medium, and tomato seedlings were gathered after about 10 d of culture. The cotyledon was cut off at both ends, and about 0.5 cm of the hypocotyl was cut, and placed on a differentiation medium for dark culture for 2 d. The appropriate constructs were transformed into these hypocotyls using *Agrobacterium tumefaciens* strain GV3101. We then utilized ½-strength MS medium supplemented with 60 mg/L kanamycin to screen for transgenic plants. After ~ 20 d, the callus grew from the explant, and was left for 2 months to sprout, then separated from the explant and inserted into rooting medium. After rooting, the cultivated seedlings were transplanted into pots containing nutrient soil for 7 d, and then to the field when adaptability was strong. Isolated kanamycin-resistant T_0_ plants were evaluated via semi-quantitative reverse transcription PCR using *LeGPA1* primers (Supplementary Table S1), and qRT-PCR was then used to confirm the identities of different transgenic plants. Plants in the T_2_-generation that retained their ability to grow on MS media supplemented with 60 mg/L kanamycin were utilized as transgenic plants in downstream assays.

### Assessment of stress response-induced changes in plants

Plants (wild-type or transgenic) from the T_2_-generation were grown for 3–6 weeks in a 25 °C incubation chamber (16/8 h light/dark cycle; 70% humidity; 200 μmol m^− 2^ s^− 1^ photon flux density). After either 3 or 6 weeks, plants with uniform sizes were subjected to 5-day cold stress exposure (4 °C). Plants were grown in three separate growth chambers for replicate samples. Changes in plant phenotypes were then visually assessed, and images were taken using a Canon 80D camera. The second and third leaves from the tops of each of these plants were additionally collected, and stress response-related and antioxidant enzyme gene expression in these leaves was assessed using qRT-PCR. These samples were also used for antioxidant activity assays and assessments of plant physiology.

### Physiological parameter analyses

Relative water content (RWC) was assessed as described in a study conducted by Lara et al. [42] as follows: RWC = (FW − DW)/(TW − DW) × 100%. In this equation, FW corresponds to leaf fresh weight, TW corresponds to turgid weight (after leaves being incubated in dH_2_O for 24 h in the presence of light), and DW corresponds to dry weight (after leaves were dried at 70 °C to a constant weight).

The levels of MDA were assessed using thiobarbituric acid reaction following Du et al. [43] with modifications: The excised leaves were pre-rinsed with dH_2_O, and the collected discs were used for the measurement of MDA level by using a spectrophotometer (UV-160A; Shimadzu Scientific Instruments, Japan).

Relative electrolyte leakage (REL) was evaluated using a conductivity meter (EC 215; Markson Science Inc., CA, USA) [43] by REL = (C1-CW)/(C2-CW) × 100, in which formula the C1 and C2 correspond to pre- and post-boiled conductivity, respectively, and the CW corresponds to dH_2_O conductivity.

The maximal efficiency of photosystem II (PSII) was assessed using a portable fluorescence analyzer (DUAL-PAM-100; Walz, Germany). The leaves were pre-processed by 30 min placing at dark, followed by 1 s flash light exposure. Variable fluorescence (F_v_) was evaluated by F_v_ = F_m_ – F_0_ [44], in which formula the F_0_ (minimal fluorescence) corresponds to the dark-adapted state with all the centers of PSII reaction opened; the F_m_ (maximal fluorescence) corresponds to the light-saturated state with all the centers of PSII reaction closed.

Free proline was assessed as in the study of Bates et al. [45]. Briefly, 4 mL of 3% sulfosalicylic acid was used to extract leaf samples (200 mg) for 10 min at 100 °C, after which homogenates were spun for 2 min at 12,000×*g*. Next, a 2-mL supernatant volume was mixed with equivalent volumes of acid-ninhydrin reagent and glacial acetic acid. This solution was then boiled for 30 min, before being transferred into an ice bath. Absorbance at 520 nm was then assessed following toluene (4 mL)-mediated extraction of the organic phase. Proline concentrations were determined relative to a proline standard curve.

The Anthrone method was used for analyses of soluble sugars, using glucose as a standard [46]. Briefly, an initial leaf sample (200 mg) was ground, homogenized in a 1 ml dH_2_O volume, boiled for 20 min, centrifuged for 10 min at 13,000×*g*, and a 2-mL supernatant volume was then combined with 1.8 mL dH_2_O and 2.0 mL of 0.14% (w/v) Anthrone solution in 100% H_2_SO_4_. This solution was rested for 20 min in boiling water, after which it was cooled, and the A_620_ was assessed. Total soluble sugar levels were assessed by comparing these A_620_ values with those derived from a glucose standard curve. Experiments were conducted in triplicate using three replicate samples.

### ROS and antioxidant activity assays

Approximately 0.5 g fresh leaves were collected from the transgenic (T_2_) or wild-type plants for exposure to stress. The minced leaf samples were homogenized in 4 ml of 50 mM sodium phosphate buffer (pH 7.8) supplemented with 10 mM β-ME and 1% polyvinylpyrrolidone, followed by centrifuging for 15 min at 4 °C and 17,426×g. The activities of CAT and SOD were measured following previously described methods [47, 48], respectively. The activity of POD was assessed using a microplate reader (Infinite M200 Pro; Tecan Group Ltd., Männedorf, Switzerland) [49].

The levels of H_2_O_2_ and O_2_^−^ were also evaluated by measuring the absorbance using the above-mentioned UV-160A spectrophotometer (Shimadzu Scientific Instruments, Japan) [50].

### Statistical analysis

Data were assessed using SPSS v13.0 and GraphPad Prism 7.0. Relative *LeGPA1* expression data are presented as mean ± standard deviation from triplicate samples, with three leaves per seedling being used as a replicate. Expression levels were normalized to baseline (0 h) levels. Dunnett’s multiple comparison test was used to compare plants. *P < 0.05 and **P < 0.01 correspond to significant and very significant, respectively.

## Discussion

The Gα subunit is an important component of the heterotrimer G-protein complex; it not only plays important roles in various plant growth and development processes but also participates in responses to abiotic stresses, such as drought and high and low temperatures [35, 51, 52]. The Gα subunit has been well studied in model plants, but it had not been previously identified in processing tomatoes. In this study, we identified a G-protein Gα subunit from processing tomatoes and studied its growth and development, as well as the resistance to low temperature it provides.

We found the *LeGPA1* gene of processing tomatoes is homologous with the *GPA1* gene of other nightshade species. Previous studies have found that *GPA1* is only expressed in mature seeds [53], and *GPA1* expression has been detected in all stages of development and all organs. *GPA1* expression is highest in the roots, followed by the stem tip, hypocotyl, cotyledon, and leaf [54], and levels are higher in immature than mature organs [53]. We analyzed the expression of *LeGPA1* in various organs of processing tomato plants and found that *LeGPA1* was expressed in all organs tested (relative expression abundance: roots > leaves > fruits > stems). Such an expression pattern suggests that *LeGPA1* is involved in the regulation of the growth and development of processing tomatoes. We also performed a subcellular localization experiment and found that *LeGPA1* is also localized in the cell membrane. This is consistent with the literature on G-proteins in plants [55–57].

The G-protein Gα subunit has been identified in Arabidopsis, rice, and maize, and acts as a positive regulator of cell proliferation and growth in all three species [21, 58, 59]. Through the functional analysis of the G-protein α subunit GPA1 in cucumber, Yan et al. found that overexpression of *CsGPA1* promoted seed germination and early seedling growth, while *CsGPA1* interference inhibited seedling growth. By testing the biological characteristics of transgenic lines and wild-type processing tomato plants [60], here we found that *LeGPA1*-overexpressing tomato plants were significantly taller than the wild-type plants, whereas the RNA interference plants were shorter. This indicates that *LeGPA1* plays an important role in the regulation of processing tomato plant height, which is consistent with what has been described for Arabidopsis, rice, and maize.

The G-protein α subunits also play important roles in the signal transduction pathway of abiotic stress in plants [26]. The full transcriptome microarray analysis of rice revealed that rice *RGA1* could regulate low temperature, salt, and drought stress and transmit stress signals to the small phosphorylase GTPase and corresponding effector proteins or molecules, such as ion channels [28]. In pea, expression of the *PsGPA1* gene was significantly altered by NaCl and high temperature [29]. A study of *Brassica napus* found that the expression of *BnGA1* was induced by four abiotic stresses [20% PEG6000, 200 mm NaCl, low temperature (4 °C), and high temperature (40 °C)] and that *BnGA1* plays an important role in resisting abiotic stress [54]. Consistent with the cited literature, here we report that in processing tomato, *LeGPA1* is induced by drought, high salt, and low temperature.

*GPA1* in Arabidopsis is involved in oxidative stress signal transduction. For example, *GPA1* can positively regulate abiotic stress factors upstream of ROS production [27]. ROS are produced and accumulate under cold stress; they destroy cells and produce MDA [61, 62]. Antioxidant enzymes are an important part of the ROS scavenging system in plant cells and, therefore, play important roles in plant cold resistance [63, 64]. For example, *Pinus koraiensis* is an evergreen tree species with strong cold resistance. A large number of DEGs were identified in *P. koraiensis* under cold stress, especially the DEGs removing ROS in antioxidation mechanisms [65]. Antioxidant enzyme activities in plants have been reported to increase under low-temperature stress, which might be due to the upregulation of corresponding genes [60, 66, 67]. In line with the literature, here we report that the expression of *LeGPA1* in processing tomatoes is up-regulated under low-temperature stress. Moreover, overexpression of *LeGPA1* in a transgenic tomato line improved the antioxidant capacity and decreases membrane lipid peroxidation under low-temperature stress, indicating that *LeGPA1* positively regulates the response of processing tomatoes to low temperature.

Multiple genes have been implicated in plant response to low-temperature stress, including ICE1-activation of the CBF pathway [68–71], trehalose 6-phosphatase (TPS) (a key enzyme in trehalose synthesis) and TPS-related genes [72, 73], and the Arabidopsis cold resistance gene 413 (*AtCOR413*) (important for frost resistance) [74]. An ICE-CBF-COR signaling pathway activates the appropriate expression of downstream genes, which encode osmoregulation substances [75]. This study analyzed the expression levels of genes related to low-temperature stress in processing tomatoes. *LeGPA1* increased *LeICE1* gene expression in processing tomatoes, thereby regulating the transcription of the *LeCBF1* gene. Thus, the expression of *LeCOR413PM2* and *LeTPS1* and its downstream regulatory gene, *LeDRCi7*, was induced. These findings identify the molecular mechanisms likely involved *LeGPA1*-mediated low-temperature tolerance in processing tomato. However, the molecular mechanisms that regulate LeGPA1 activation and inactivation, as well as the downstream effector cycling, are still unknown. Therefore, to elucidate how plant G proteins interact with environmental signals to maintain stable growth and development in a varied environment, in the future, molecular biological technologies (e.g., immunoco-precipitation and bilateral fluorescence complementary) should be further utilized to verify, isolate, and identify the proteins interacting with LeGPA1, and determine the downstream effector molecules and signal transduction mechanism of LeGPA1.

## Conclusion

Together, these findings suggest that *LeGPA1* overexpression is a viable approach to improving resistance to cold stress in tomato. We found that *LeGPA1* overexpression is associated with increased cell membrane integrity and stability in response to cold stress, while also reducing the accumulation of membrane lipid peroxidation products, decreasing the ion leakage rates, and increasing antioxidant enzyme activity. These changes were associated with improved photochemical electron transport efficiency and antioxidant enzyme activity. For example, the *LeGPA1*-overexpressing plants exhibited reduced ROS levels compared with the wild-type controls. Low-temperature tolerance was improved in these plants via the maintenance of elevated permeable proline and soluble sugar levels to protect against cellular damage.

## Supplementary information

**Additional file 1: Figure S1.** The acquirements of *LeGPA1*-OE and *LeGPA1*-RNAi lines. **Figure S2.** Semi-quantitative PCR was used to assess *LeGPA1* transcript levels in leaves collected from plants grown under standard conditions. M: DL5000 DNA Marker, WT: Wild-type tomato plants, O1-O3: *LeGPA1*-overexpressing transgenic tomato plants, RI-R3: RNAi transgenic tomato plant lines. **Supplementary Table S1.** List of primers used in this study.

## Data Availability

The datasets used and/or analysed during the current study are available from the corresponding author on reasonable request.

## References

[1] Shabala S, Bose J, Fuglsang AT, Pottosin I (2015). On a quest for stress tolerance genes: membrane transporters in sensing and adapting to hostile soils. J Exp Bot.

[2] Ji TH, Grossmann M, Ji I (1998). G-protein coupled receptors. J Biol Chem.

[3] Burg JS, Ingram JR, Venkatakrishnan AJ, Jude KM, Dukkipati A, Feinberg EN, Angelini A, Waghray D, Dror RO, Ploegh HL, Garcia KC (2015). Structural basis for chemokine recognition and activation of a viral G protein-coupled receptor. Science..

[4] Wettschureck N, Offermanns S (2005). Mammalian G proteins and their cell type specific functions. Physiol Rev.

[5] Assmann SM (2002). Heterotrimeric and unconventional GTP binding proteins in plant cell signaling. Plant Cell.

[6] Ma H, Yanofsky MF, Meyerowitz EM (1990). Molecular cloning and characterization of GPA1, a G protein alpha subunit gene from *Arabidopsis thaliana*. Proc Nati Acad Sci..

[7] Ma H (1995). GTP-binding proteins in plants: new members of an old family. Plant Mol Biol.

[8] Ishikawa A, Tsubouchi H, Iwasaki Y, Asahi T (1995). Molecular-cloning and characterization of a cDNA for the alpha-subunit of a G-protein from rice. Plant Cell Physiol..

[9] Seo HS, Kim HY, Jeong JY, Lee SY, Bahk JD (1995). Molecular cloning and characterization of RGA1 encoding a G protein α subunit from rice (*Oryza sativa* l. ir-36). Plant Mol Biol.

[10] Jones HD, Smith SJ, Desikan R, Plakidou-Dymock S, Hooley LR (1998). Heterotrimeric G proteins are implicated in gibberellin induction of α-amylase gene expression in wild *Oat aleurone*. Plant Cell.

[11] Ma H, Yanofsky MF, Huang H (1991). Isolation and sequence analysis of TGA1 cDNAs encoding a tomato G protein α subunit. Gene..

[12] Gotor C, Lam E, Cejudo FJ, Romero LC (1996). Isolation and analysis of the soybean *SGA2* gene (cDNA), encoding a new member of the plant G-protein family of signal transducers. Plant Mol Biol.

[13] Kim WY, Cheong NE, Lee DC, Je DY, Bahk JD, Cho MJ (1995). Cloning and sequencing analysis of a full-length cDNA encoding a G protein α subunit, SGA1, from soybean. Plant Physiol.

[14] Marsh JF, Kaufman LS (1999). Cloning and characterization of PGA1 and PGA2: two G protein α-subunits from pea that promote growth in the yeast *Saccharomyces cerevisiae*. Plant J.

[15] Perroud PF, Diogon T, Crevecoeur M, Greppin H (2000). Molecular cloning, spatial and temporal characterization of pinach SOGA1 cDNA, encoding an α subunit of G protein. Gene..

[16] Kaydamov C, Tewes A, Adler K, Manteuffel R (2000). Molecular characterization of cDNAs encoding G protein alpha and beta subunits and study of their temporal and spatial expression patterns in *Nicotiana plumbaginifolia* viv. Biochim Biophys Acta.

[17] Ullah H, Chen JG, Wang S, Jones AM (2002). Role of a heterotrimeric G protein in regulation of Arabidopsis seed germination. Plant Physiol.

[18] Chen YL, Huang R, Xiao YM, Lü P, Chen J, Wang XC (2004). Extracellular calmodulin-induced stomatal closure is mediated by heterotrimeric G protein and H_2_O_2_. Plant Physiol.

[19] Pandey S, Che JG, Jones AM, Assmann SM (2006). G-protein complex mutants are hypersensitive to abscisic acid regulation of germination and postgermination development. Plant Physiol.

[20] Ullah H, Chen JG, Temple B, Boyes DC, Alonso JM, Davis KR (2003). The β-subunit of the Arabidopsis G-protein negatively regulates auxin-induced cell division and affects multiple developmental processes. Plant Cell.

[21] Ullah H, Chen JG, Young JC, Im KH, Sussman MR, Jones AM (2001). Modulation of cell proliferation by heterotrimeric G-protein in Arabidopsis. Science..

[22] Jones AM, Ecker JR, Chen JG (2003). A reevaluation of the role of the heterotrimeric G protein in coupling light responses in Arabidopsis. Plant Physiol.

[23] Ashikari M, Wu J, Yano M, Sasaki T, Yoshimura A (1999). A rice gibberellin-insensitive dwarf mutant gene *dwarf 1* encodes the α-subunit of GTP-binding protein. Proc Nati Acad Sci USA.

[24] Fujisawa Y, Kato T, Ohki S, Ishikawa A, Kitano H, Sasaki T (1999). Suppression of the heterotrimeric G protein causes abnormal morphology, including dwarfism, in rice. Proc Nati Acad Sci.

[25] Oki K, Inaba N, Kitagawa K, Fujioka S, Iwasaki Y (2008). Function of the subunit of rice heterotrimeric G protein in brassinosteroid signaling. Plant Cell Physiol.

[26] Yadav DK, Shukla D, Tuteja N (2013). Rice heterotrimeric g protein alpha subunit (RGA1): in silico analysis of the gene and promoter and its upregulation under abiotic stress. Plant Physiol Biochem.

[27] Zhang W, Jeon BW, Assmann SM (2011). Heterotrimeric G-protein regulation of ROS signalling and calcium currents in Arabidopsis guard cells. J Exp Bot.

[28] Jangam AP, Pathak RR, Raghuram N (2016). Microarray analysis of rice *d1* (RGA1) mutant reveals the potential role of G-protein alpha subunit in regulating multiple abiotic stresses such as drought, salinity, heat, and cold. Front Plant Sci.

[29] Misra S, Wu Y, Venkataraman G, Sopory SK, Tuteja N (2007). Heterotrimeric G-protein complex and G-protein-coupled receptor from a legume (*Pisum sativum*): role in salinity and heat stress and cross-talk with phospholipase c. Plant J.

[30] Ferrero-Serrano A, Assmann SM (2016). The α-subunit of the rice heterotrimeric G protein, RGA1, regulates drought tolerance during the vegetative phase in the dwarf rice mutant *d1*. J Exp Bot.

[31] Chakraborty N, Sharma P, Kanyuka K, Pathak RR, Choudhury D, Hooley R (2015). G-protein α-subunit (GPA1) regulates stress, nitrate and phosphate response, flavonoid biosynthesis fruit/seed development and substantially shares GCR1 regulation in *A. thaliana*. Plant Mol Biol.

[32] Chakraborty N, Singh N, Kaur K, Raghuram N (2015). G-protein signaling components GCR1 and GPA1 mediate responses to multiple abiotic stresses in Arabidopsis. Front Plant Sci.

[33] Yadav DK, Islam SM, Tuteja N (2012). Rice heterotrimeric g protein gamma subunits (RGG1 and RGG2) are differentially regulated under abiotic stress. Plant Signal Behav.

[34] Yadav DK, Shukla D, Tuteja N (2014). Isolation, in silico characterization, localization and expression analysis of abiotic stress-responsive rice G-protein β subunit (RGB1). Plant Signal Behav.

[35] Ma Y, Dai X, Xu Y, Luo W, Zheng X, Zeng D (2015). Cold1 confers chilling tolerance in rice. Cell..

[36] Thompson PA (1974). Characterisation of the germination responses to temperature of vegetable seeds. I. Tomatoes. Sci Hortic.

[37] Lovdal T, Lillo C (2009). Reference gene selection for quantitative real-time PCR normalization in tomato subjected to nitrogen, cold, and light stress. Anal Biochem.

[38] Livak KJ, Schmittgen TD (2001). Analysis of relative gene expression data using real-time quantitative PCR and the 2-△△Ct method. Methods..

[39] Nelson BK, Cai X, Nebenfuhr A (2007). A multi-color set of in vivo organelle markers for colocalization studies in Arabidopsis and other plants. Plant J.

[40] Hu W, Yuan Q, Wang Y, Cai R, Deng X, Wang J (2012). Overexpression of a wheat aquaporin gene, *TaAQP8*, enhances salt stress tolerance in transgenic tobacco. Plant Cell Physiol..

[41] Zhou S, Hu W, Deng X, Ma Z, Chen L, Huang C (2012). Overexpression of the wheat aquaporin gene, *TAAQP7*, enhances drought tolerance in transgenic tobacco. PLoS One.

[42] Lara MV, Disante KB, Podestá FE, Andreo CS, Drincovich MF (2003). Induction of a crassulacean acid like metabolism in the C_4_ succulent plant, *Portulaca oleracea* L.: physiological and morphological changes are accompanied by specific modifications in phosphoenolpyruvate carboxylase. Photosynth Res.

[43] Du Z, Bramlage WJ (1992). Modified thiobarbituric acid assay for measuring lipid oxidation in sugar-rich plant tissue extracts. J Agric Food Chem.

[44] Krause GH, Weis E (1991). Chlorophyll fluorescence and photosynthesis: the basics. Annu Rev Plant Physiol Mol Biol.

[45] Bates LS, Waldren RP, Teare ID (1973). Rapid determination of free proline for water-stress studies. Plant Soil.

[46] Fukao T, Xu K, Ronald PC, Bailey-Serres J (2006). A variable cluster of ethylene response factor-like genes regulates metabolic and developmental acclimation responses to submergence in rice. Plant Cell.

[47] Cakmak I, Marschner H (1992). Magnesium deficiency and high light intensity enhance activities of superoxide dismutase, ascorbate peroxidase, and glutathione reductase in bean leaves. Plant Physiol.

[48] Beauchamp C, Fridovich I (1971). Superoxide dismutase: improved assays and an assay applicable to acrylamide gels. Anal Biochem.

[49] Doerge DR, Divi IL, Churchwell ML (1997). Identification of the colored guaiacol oxidation product produced by peroxidases. Anal Biochem.

[50] Benikhlef L, Haridon LF, Abou-Mansour E, Serrano M, Binda M, Costa A (2013). Perception of soft mechanical stress in Arabidopsis leaves activates disease resistance. BMC Plant Biol.

[51] Wang XQ, Ullah H, Jones AM, Assmann SM (2001). G protein regulation of ion channels and abscisic acid signaling in Arabidopsis guard cells. Science..

[52] Coursol S, Fan LM, Le Stunff H, Spiegel S, Gilroy S, Assmann SM (2003). Sphingolipid signalling in *Arabidopsis* guard cells involves heterotrimeric G proteins. Nature..

[53] Catherine AW, Huang H, Ma H (1993). Immunolocalization of the G protein α subunit encoded by the *GPA1* gene in *Arabidopsis*. Plant Cell.

[54] Gao Y, Li T, Liu Y, Ren C, Zhao Y, Wang M (2010). Isolation and characterization of gene encoding G protein α subunit protein responsive to plant hormones and abiotic stresses in *Brassica napus*. Mol Biol Rep.

[55] Pandey S, Assmann SM (2004). The Arabidopsis putative G protein-coupled receptor GCR1 interacts with the G protein α subunit GPA1 and regulates abscisic acid signaling. The Plant Cell Online.

[56] Zhang LY, Fang KF, Lin JX (2005). Heterotrimeric G protein α-subunit is localized in the plasma membrane of *Pinus bungeana* pollen tubes. Plant Sci.

[57] Chen Y, Ji FF, Xie H, Liang JS, Zhang JH (2006). The regulator of G protein signaling proteins involve in sugar and abscisic acid signaling in Arabidopsis seed germination. Plant Physiol.

[58] Ueguchi-Tanaka M, Fujisawa Y, Kobayashi M, Ashikari M, Iwasaki Y, Kitano H (2000). Rice dwarf mutant *d1*, which is defective in the alpha subunit of the heterotrimeric G protein, affects gibberellin signal transduction. Proc Nati Acad Sci..

[59] Bommert P, Je BI, Goldshmidt A, Jackson D (2013). The maize Gα gene COMPACT PLANT 2 functions in CLAVATA signalling to control shoot meristem size. Nature..

[60] Yan Y, Zhang W, Li Y, He C, Gao L, Yu X (2018). Functions of *CsGPA1* on the hypocotyl elongation and root growth of cucumbers. Sci Rep.

[61] Achard P, Gong F, Cheminant S, Alioua M, Hedden P, Genschik P (2008). The cold-inducible CBF1 factor-dependent signaling pathway modulates the accumulation of the growth-repressing della proteins via its effect on gibberellin metabolism. The Plant Cell Online..

[62] Swain DM, Sahoo RK, Srivastava VK, Tripathy BC, Tuteja R, Tuteja N (2016). Function of heterotrimeric G-protein γ subunit RGG1 in providing salinity stress tolerance in rice by elevating detoxification of ROS. Planta..

[63] Ahmad P, Jaleel CA, Salem MA, Nabi G, Sharma S (2010). Roles of enzymatic and nonenzymatic antioxidants in plants during abiotic stress. Crit Rev Biotechnol.

[64] Liu L, Duan L, Zhang J, Zhang Z, Mi G, Ren H (2010). Cucumber (*Cucumis sativus* L.) over-expressing cold-induced transcriptome regulator ICE1 exhibits changed morphological characters and enhances chilling tolerance. Sci Hortic.

[65] Wang F, Chen S, Liang D, Qu GZ, Chen S, Zhao X (2020). Transcriptomic analyses of *Pinus koraiensis* under different cold stresses. BMC Genomics.

[66] Baek KH, Skinner DZ (2003). Alteration of antioxidant enzyme gene expression during cold acclimation of near-isogenic wheat lines. Plant Sci.

[67] Soltész A, Tímár I, Vashegyi I, Toth B, Kellos T, Szalai G, Vágújfalvi A, Kocsy G, Galiba G (2011). Redox changes during cold acclimation affect freezing tolerance but not the vegetative/ reproductive transition of the shoot apex in wheat. Plant Biol.

[68] Winfield MO, Lu C, Wilson ID, Coghill JA, Edwards KJ (2010). Plant responses to cold: transcriptome analysis of wheat. Plant Biotechnol J.

[69] Chinnusamy V, Zhu J, Zhu JK (2007). Cold stress regulation of gene expression in plants. Trends in Plant Sci.

[70] Zhu JK (2016). Abiotic stress signaling and responses in plants. Cell..

[71] Miura K, Shiba H, Ohta M, Kang SW, Sato A, Yuasa T (2012). SlICE1 encoding a MYC-type transcription factor controls cold tolerance in tomato, *Solanum lycopersicum*. Plant Biotechnol..

[72] Tomikubo Y, Yuasa T, Iwaya-Inoue M (2007). Analysis of chilling-induced trehalose-6-phosphate synthase (TPS) in tomato plants. Cryobiol Cryotechnol Japanese.

[73] Nakamura J, Yuasa T, Huong TT, Harano K, Tanaka S, Iwata T (2011). Rice homologs of inducer of CBF expression (osice) are involved in cold acclimation. Plant Biotechnol.

[74] Breton G, Danyluk J, Charron JB, Sarhan F (2003). Expression profiling and bioinformatic analyses of a novel stress-regulated multi spanning transmembrane protein family from cereals and *Arabidopsis*. Plant Physiol.

[75] Ritonga FN, Chen S (2020). Physiological and molecular mechanism involved in cold stress tolerance in plants. Plants..

