# Supplementary material for: Heterotrimeric G-protein α subunit (LeGPA1) confers cold stress tolerance to processing tomato plants (Lycopersicon esculentum Mill)
Source: BMC Plant Biol. 2020 Aug 26;20:394. doi: 10.1186/s12870-020-02615-w (PMC7448358; doi:10.1186/s12870-020-02615-w)
Supplement: Supplementary file 1 — Additional file 1: Figure S1. The acquirements of LeGPA1-OE and LeGPA1-RNAi lines. Figure S2. Semi-quantitative PCR was used to assess LeGPA1 transcript levels in leaves collected from plants grown under standard conditions. M: DL5000 DNA Marker, WT: Wild-type tomato plants, O1-O3: LeGPA1-overexpressing transgenic tomato plants, RI-R3: RNAi transgenic tomato plant lines. Supplementary Table S1. List of primers used in this study. [file 12870_2020_2615_MOESM1_ESM.docx]

Supplementary Material

**Heterotrimeric G-protein α subunit (*LeGPA1*) confers cold stress tolerance in Processing tomato plants (*Lycopersicon esculentum* Mill)**

Xinyong Guo^1^, Juju Li^1^, Li Zhang^1^, Zhanwen Zhang, Ping He, Wenwen Wang, Mei Wang, Aiying Wang, Jianbo Zhu^*^

College of Life Science, Shihezi University, Shihezi 832000, China

1 These authors contributed equally and are co-first authors: Xinyong Guo, Juju Li, Li Zhang

*Corresponding author: Jianbo Zhu

College of Life Science, Shihezi University, Shihezi 832000, China

Email: [jianboz9@sina.com](mailto:jianboz9@sina.com)

## Supplementary Figure


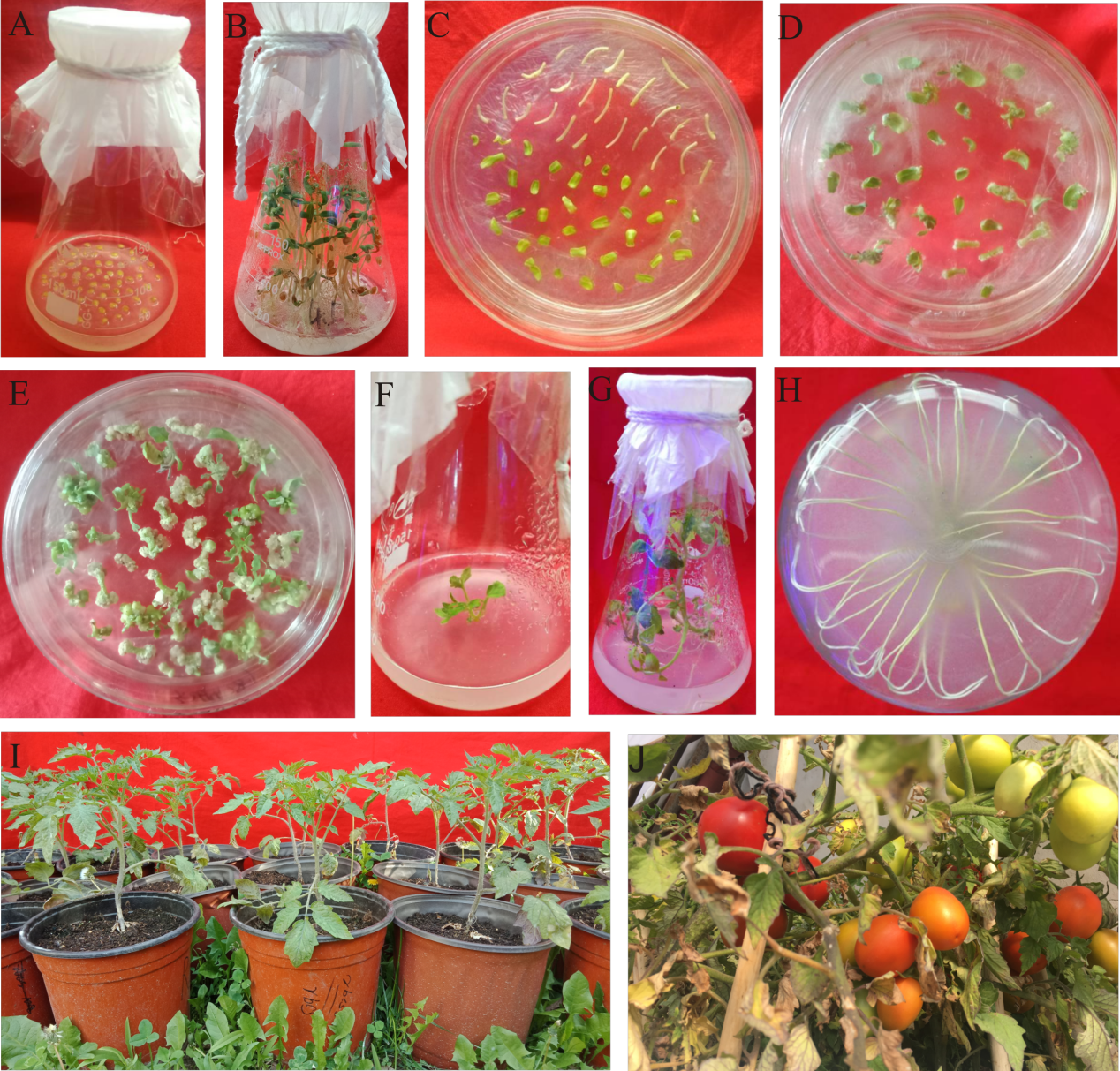


**Fig S1. The acquirements of *LeGPA1*-OE and *LeGPA1*-RNAi lines**

**a** Planting of tomato seeds. **b** Tomato seedlings. **c** Tomato explants. **d** The explants form a callus. **e** The differentiation period of adventitious tissue culture buds. **f**–**h** Rooting screening culture period. **i** Transplanting of transgenic tomato to flowerpots. **j** Transgenic tomatoes transplanted into the field.


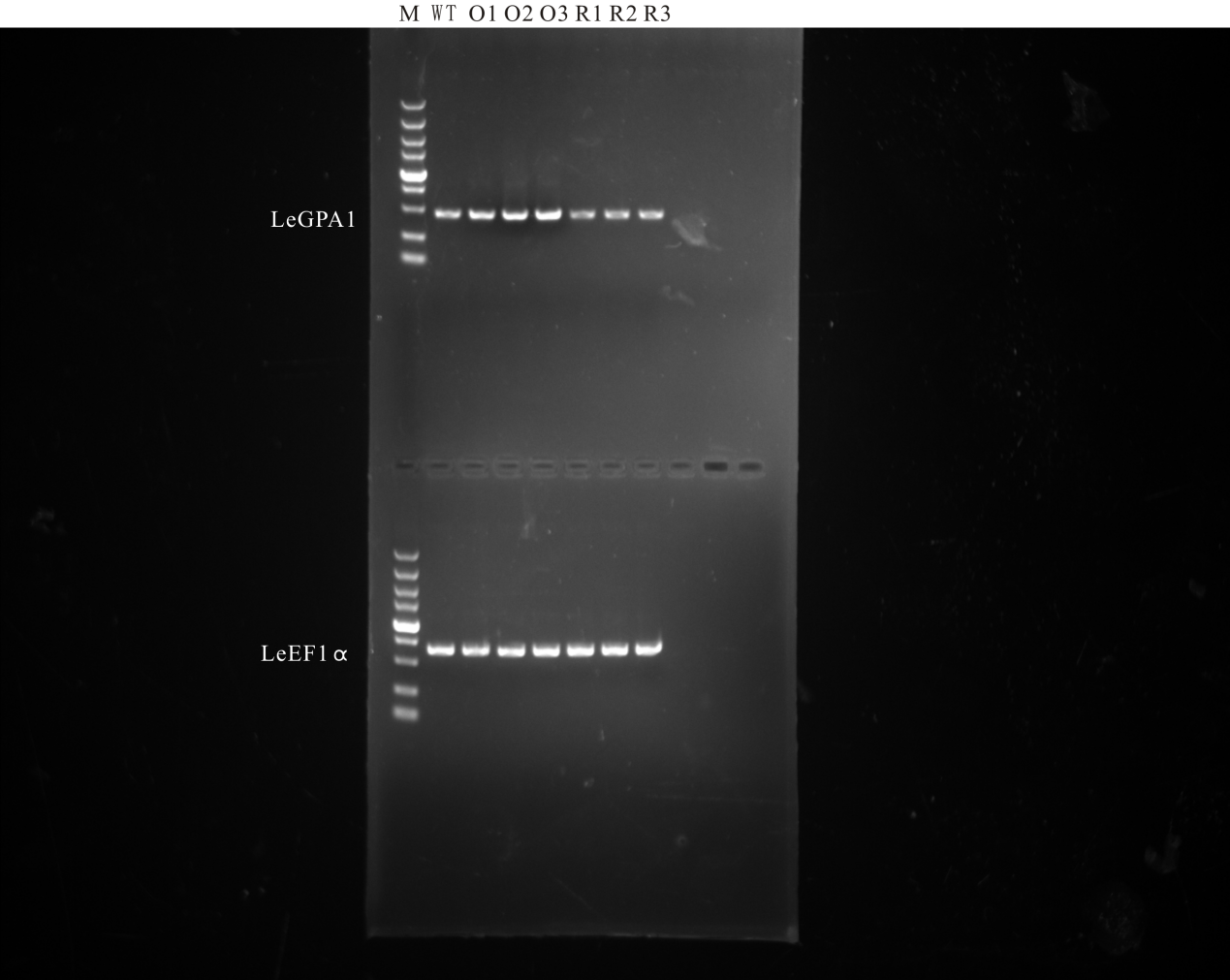


**Fig S2. Semi-quantitative PCR was used to assess *LeGPA1* transcript levels in leaves collected from plants grown under standard conditions.** M: DL5000 DNA Marker, WT: Wild-type tomato plants, O1-O3: *LeGPA1*-overexpressing transgenic tomato plants, RI-R3: RNAi transgenic tomato plant lines.

## Supplementary Table

**Supplementary Table S1.** **List of primers used in this study.**

| **Name** | **Sequence (5′-3′)** | **Purpose** | **Annealing**  **temperatures** | **Amplicon**  **size (bp)** |
| --- | --- | --- | --- | --- |
| *LeGPA1* (*Kpn* Ⅰ)-F | GGTACCATGCTGTCGGTGGTTTTCGAA | Cloning | 56℃ | 1191bp |
| *LeGPA1* (*Sal* Ⅰ)-R | GTCGACTCATAATAAACCTGCTTCGAA | Cloning |  |  |
| LeGPA1(BamHⅠ)-F | GGTACCATGCTGTCGGTGGTTTTCGAA | Subcellular localization | 56℃ | 1188bp |
| *LeGPA1* (*Xba* Ⅰ)-R | TCTAGAAATAAACCTGCTTCGAAGAGA | Subcellular localization |  |  |
| *LeGPA1* (*Xho* I and *Sal* I )-F | CTCGAGTCCAGATTGTGCCCATTA | RNAi upstream | 56℃ | 346bp |
| *LeGPA1* (*Bgl* II and *BamH* I )-R | AGATCTGACCCACTCAAAGAGTT | RNAi downstream |  |  |
| *LeGPA1-*qF | CTACAGTCAAGCCGATGATGAG | qRT-PCR | 60℃ | 172bp |
| *LeGPA1-*qR | AAAGCCAGTTTGGAACAAGAGT | qRT-PCR |  |  |
| *LeEF1α-*qF | GGAACTTGAGAAGGAGCCTAAG | qRT-PCR | 60℃ | 158bp |
| *LeEF1α-*qR | CAACACCAACAGCAACAGTCT | qRT-PCR |  |  |
| *LeSOD-*qF | GGCCAATCTTTGACCCTTT | qRT-PCR | 60℃ | 182bp |
| *LeSOD-*qR | AGTCCAGGAGCAAGTCCAGT | qRT-PCR |  |  |
| *LePOD-*qF | GTCCGGGAGTTGTTTCTTGT | qRT-PCR | 60℃ | 140bp |
| *LePOD-*qR | ATCACCATTGGCTTCTGACA | qRT-PCR |  |  |
| *LeCAT-*qF | ATTTGGTGGAGAAACTTGCC | qRT-PCR | 60℃ | 152bp |
| *LeCAT-*qR | CTGTACACCAGGAGCTCGAA | qRT-PCR |  |  |
| *LeDRCi7-*qF | TTGTGTTTCTGTGTTGTTTTGG | qRT-PCR | 60℃ | 109bp |
| *LeDRCi7-*qR | GCACATACATATGCACTTACATACAG | qRT-PCR |  |  |
| *LeTPS1-*qF | GGTACCTGCAGACACTGAGTGGAA | qRT-PCR | 60℃ | 177bp |
| *LeTPS1-*qR | CTGTCGACTATACAAAGGATGCATGATTCTTAAC | qRT-PCR |  |  |
| *LeICE1-*qF | GGAAGGAAAAGCGGTGAAC | qRT-PCR | 60℃ | 144bp |
| *LeICE1-*qR | AACACATCCAACACAAACCC | qRT-PCR |  |  |
| *LeCBF1-*qF | TTCATCGTCATCGTCGTTTTCT | qRT-PCR | 60℃ | 125bp |
| *LeCBF1-*qR | TCCTCTTCCTGATTCCCCTGT | qRT-PCR |  |  |
| *LeCOR413PM2-*qF | AACTGGAGGAGCAACATA | qRT-PCR | 60℃ | 164bp |
| *LeCOR413PM2-*qR | TCAAGCCAATCTGGAAAG | qRT-PCR |  |  |
| *LeGPA1-*qF | AGGTTCCAGATTGTGCCCATTA | RT-PCR | 60℃ | 472bp |
| *LeGPA1-*qR | TCCTGTTGAAACTGACTGGTAATCT | RT-PCR |  |  |
| *LeEF1α-*qF | TCAGGCTGACTGTGCTGTTCTC | RT-PCR | 60℃ | 644bp |
| *LeEF1α-*qR | CTGGGTCATCCTTGGAGTTTGAG | RT-PCR |  |  |
